# Advancing research to eliminate mental illness stigma: an interventional study to improve community attitudes towards depression among University students in Singapore

**DOI:** 10.1186/s12888-021-03106-4

**Published:** 2021-02-18

**Authors:** Chong Min Janrius GOH, Shazana SHAHWAN, Jue Hua LAU, Wei Jie ONG, Gregory Tee Hng TAN, Ellaisha SAMARI, Kian Woon KWOK, Mythily SUBRAMANIAM, Siow Ann CHONG

**Affiliations:** 1 Research Division, Institute of Mental Health, Buangkok Green Medical Park, 10 Buangkok View, Singapore, 539747 Singapore; 2 School of Social Sciences, Nanyang Technological University, 50 Nanyang Avenue, Singapore, 639798 Singapore

**Keywords:** Anti-stigma intervention, Dperession, Attitudes, University students, CAMI, Singapore, ARTEMIS

## Abstract

**Background:**

After decades of anti-stigma initiatives, the Advancing Research To Eliminate Mental Illness Stigma (ARTEMIS) intervention study is one of the first in Singapore to evaluate the effects of an anti-stigma intervention on attitudes towards depression in university students.

**Methods:**

390 university students from a local university in Singapore were voluntarily recruited for the study. The ARTEMIS intervention comprises an educational and social contact component, as well as a question and answer (Q&A) session with experts in the area of mental health. The Community Attitudes towards Mental Illness (CAMI) scale was administered at baseline, post-intervention and at 3-months follow-up. A confirmatory factor analysis (CFA) was conducted.

**Results:**

The CFA identified a 3-factor model for the CAMI with a decent fit (RMSEA = 0.06, CFI = 0.93, TLI = 0.93, SRMR = 0.06). Favourable shifts in attitudes across the factors were observed immediately after the intervention (*p* <  0.001). Gender (β = − 1.19, 95% CI: − 2.10, − 0.27, *p* = 0.01) and nationality (β = − 1.23, 95% CI: − 2.35, − 0.11, *p* = 0.03) were identified as significant correlates for the community mental health ideology (CMHI) factor. Linear effects indicated that having a close social contact with mental illness observed a smaller decrease in authoritarianism scores from pre- to post-intervention (β = 0.85, 95% CI: 0.18, 1.53, *p* = 0.01); whereas quadratic effects found a greater decrease in scores from post-intervention to after 3-months for benevolence (β = − 0.34, 95% CI: − 0.52, − 0.16, *p* <  0.001) and CMHI (β = − 0.22, 95% CI: − 0.45, − 0.002, *p* = 0.048).

**Conclusion:**

The anti-stigma intervention shows promising short-term results across the CAMI dimensions even after adjusting for sociodemographic correlates. However, the intervention did not observe the sustained attitude shifts after 3-months. Recommendations for future anti-stigma interventions were also considered.

## Background

A nationwide mental health literacy study reported that people with mental illness (PMI) in Singapore face a considerable amount of stigma [1]. Mental illness stigma is a complex social phenomenon that often negatively impacts individuals experiencing mental illness. The seminal conceptualisation of stigma by Goffman is typically adopted and defined as a discrediting attribute that carries a mark of shame and greatly reduces social value [2, 3]. One of the many consequences of stigmatising attitudes towards mental illness is the impact it has on help-seeking and recovery [4]. For instance, compared to other types of help-seeking barriers, stigma was ranked the fourth highest in a systematic review that included both quantitative and qualitative studies [5]. People with depression facing these stigmatising attitudes, from the public or experiencing self-stigma, could delay treatment leading to a longer duration of untreated illness; which could have severe consequences such as poorer treatment response, lower rates of remission, increased rates of chronicity and increased frequency of relapse [6].

Psychiatric disorders are prevalent in Singapore’s population (13.9%), especially among those aged 18–34 years (21.6%) [7]. Youths were thus identified as a vulnerable group in the Singapore population, who were at a higher risk of developing mental health issues [8]. In line with international findings, Vaingankar et al. established that psychiatric illnesses typically present during young adulthood in Singapore [9–11]. Furthermore, individuals who were tertiary educated were less likely to seek treatment for their mental disorders [7].

A significant milestone in a young person’s life is when they matriculate to university, whereby they gain more independence from their family. During this period, youths are exposed to a plethora of psychosocial risk factors (e.g. pressures to succeed academically and loneliness) that could potentially lead to higher risks of major depressive disorder (MDD) and/or generalised anxiety disorder (GAD) [12]. In addition to the clinical ramifications of these debilitating conditions, university students could suffer consequences in their academic performance which in turn would greatly impact their socioeconomic opportunities (i.e. employment, career, marital status) later in life as well [4]. By targeting university students, an intervention could potentially foster an attitude of caring towards PMI among young adults who are likely to carry it throughout their adult lives. Hence, it is imperative that the gap in knowledge, and negative attitudes and behaviours towards PMI be addressed among university students, specifically, by cultivating greater understanding about mental health as well as encouraging help-seeking and mutual support among peers and community.

In an effort to alleviate mental illness stigma, short-term interventions that specifically targetted university students have been developed throughout the years, [13]. The Advancing Research to Eliminate Mental Illness Stigma (ARTEMIS) intervention was developed in-house, by incorporating two key components that Corrigan and Penn stated in their theory of stigma reduction – education and contact [14]. The core elements of their theory takes into consideration Thornicroft et al.’s conceptualisation of stigma, in which addressing issues related to 1) a lack of knowledge about mental health; 2) negative attitudes; 3) rejecting and avoidant behaviours towards PMI, could help alleviate stigmatising attitudes [15]. A study by Pang et al. reported that youths in Singapore carry with them several misconceptions of mental illnesses, as well as issues of disclosure and fear of being stigmatised themselves [16]. The education component of an anti-stigma intervention seeks to provide accurate information and enlighten individuals about the myths and stereotypes that surround depression, and this has been demonstrated as an effective method of reducing personal stigma [17]. Treatment options and information on sources of help are essential educational content for improving attitudes towards the use of mental health services [18]. The inclusion of direct contact with someone who has had a history of mental illness has also demonstrated effectiveness in modifying negative attitudes towards individuals with mental illnesses [17].

Early 1990s saw the rise of advocation for greater awareness of mental illness led by the Institute of Mental Illness (IMH) and the Singapore Association for Mental Health [19]. Roughly over the next two decades (2000s to mid-2010s), other organisations such as the Community Health Assessment Team (CHAT), Samaritans of Singapore (SOS) and the National Council of Social Service (NCSS) fostered a network of governmental and community agencies to reduce the stigma of mental illness [19]. However, despite decades of locally executed anti-stigma programmes and campaigns to educate and raise awareness about mental illness, PMI in Singapore still face a significant amount of stigma. As commented by Kuek et al., an extensive literature search on evaluative studies on anti-stigma programmes between the years 2000 and 2019 found no peer-reviewed published articles on any anti-stigma programmes in Singapore [19]. To the best of the authors’ knowledge, no similar intervention has been developed or evaluated in Singapore. And that the ARTEMIS intervention while similar to those conducted elsewhere such as Ahuja et al. and Friedrich et al. [20, 21], was locally adapted to address concerns of youth in Singapore as well as give them detailed information on help-seeking in the local context. Therefore, the purpose of this study is to evaluate the effectiveness of an anti-stigma intervention towards depression on the community attitudes towards mental illness (CAMI) factors as well as to observe any sustained effects after 3-months; to locally validate the CAMI among university students; and to examine the sociodemographic correlates of the CAMI among university students in Singapore.

## Methods

### Study design and sampling procedure

This is a single-arm interventional pre-post study that includes a follow-up period of 3-months (via QuestionPro, an online survey platform). The Advancing Research To Eliminate Mental Illness Stigma (ARTEMIS) was delivered as a single interventional session with 9 sequential sessions conducted to accommodate all the students. Each session had a maximum of 50–80 students (dependent on the size of the venue) and the sessions were conducted over a period of 6 months (October 2018 to April 2019). Each session ran approximately for 50 min, in the evening after classes, at an available venue of the collaborating local university’s campus (i.e. a lecture hall or dormitory recreation room). The intervention comprises: 1) an educational component that includes a lecture on depression, supplemented with a PowerPoint presentation and the WHO “I had a black dog, his name was depression” video [22], conducted by a trained research clinical psychologist. The 30 min PowerPoint-assisted lecture introduced the participants to the common symptoms, both global and national prevalence, help-seeking and treatment avenues, and the biopsychosocial aetiology of depression; 2) a 10 min contact component conducted by a person with lived experience of mental illness, whereby she shared the clinical aspects of depression, the challenges she faced, as well as her journey towards recovery. The person ensured that the narrative was similar in all the sessions; 3) a 10 min question and answer (Q&A) session with a senior consultant psychiatrist, a mental health research expert as well as the person with lived experience. This provided an opportunity where participants could clarify and solicit more information pertaining to the presentations. A detailed account of the ARTEMIS intervention can be found in Subramaniam et al. and Shahwan et al. [23, 24]. Data of the ARTEMIS intervention was collected using questionnaires at baseline (pre-intervention), immediately after the intervention (post-intervention), and at 3-months follow-up. The questionnaires were delivered in English, the administrative language in Singapore. Prior to participation, the request for informed consent was sent by email to the university students or for their parents/guardians if they were under 21 years of age, which is the age of majority in Singapore. On the day of the session, informed written consent was obtained by a research staff. Consent was collected from all students before they participated in the intervention. Approval for the ARTEMIS study was given by the institutional ethics committee of The National Healthcare Group, Domain Specific Review Board. The local university is home to several colleges and schools with approximately 33,000 undergraduate and postgraduate students across a diversity of disciplines ranging from STEM to humanities, and the arts. Convenience sampling was employed for the ARTEMIS study, where an email invitation to the study was sent to students at the collaborating local university and posters were placed at strategic campus locations to advertise the ARTEMIS study. Inclusion criteria for this study included: 1) a current student of the university at the time of recruitment; 2) aged 18 to 35 years, 3) able and willing to provide informed consent; for students aged below 21 years, parental consent was required for participation, and 4) literate in English.

### Measures & outcomes

#### Community attitudes towards mental illness (CAMI)

The CAMI consists of 40 statements with a 5-point Likert-scale that ranges from “1 = Strongly Agree” to “5 = Strongly Disagree” to systematically gain insights to the communities’ attitudes towards individuals with mental illness. The CAMI originally measures 4 factors: 1) Authoritarianism, 2) Benevolence, 3) Social Restrictiveness, and 4) Community Mental Health Ideology. The statements of the CAMI expressed 5 pro- and 5 anti-sentiments for each dimension (i.e. 4 sets of 10 statements). For instance, the statement “As soon as a person shows signs of mental disturbance, he should be hospitalized” represents a pro-authoritarianism, whereas “The mentally ill should not be treated as outcasts of society” indicates anti-authoritarianism attitudes. Reverse scoring was done for the anti-sentiment statements for each dimension. Authoritarianism reflects the community’s view that individuals with mental illness are of an inferior class that requires coercive methods to manage them. Social Restrictiveness indicates the view that individuals with mental illness pose a threat to the community. Benevolence represents sympathetic views based on humanistic and religious principles. The Community Mental Health Ideology refers to the values associated with acceptance of having PMI integrated into the community as being therapeutic. Additionally, it also inquiries into the impact of having mental health facilities in residential areas. Hence, higher scores on both Authoritarianism and Social Restrictiveness would suggest higher unfavourable attitudes, whereas endorsing (i.e. higher scores) Benevolence and Community Mental Health ideology would indicate more supportive and inclusive attitudes towards individuals with mental illness. Refer to the confirmatory factor analysis (CFA) for the factor structure used in this study.

#### Sociodemographic information

Participants provided information on age, sex, nationality, and ethnicity. Due to the under-representation of the ethnic-minority groups, Malay, Indian and other ethnicities were subsumed into a single category (i.e. Others) and compared to Chinese ethnicity. For nationality, responses from Singapore citizens and Permanent Residents (PR) were examined as one category versus participants with student visas. The following measures inquired on the participants’ exposure to mental illness such as “Do you have a close friend or family member who has a mental illness?” and “Do you have past experience within the mental health field?” whereby possible answers were “yes” or “no”, for both.

### Statistical analysis

Statistical analysis was performed using MPlus version 8.2 and SPSS version 22. Frequencies and percentages were calculated for categorical variables, whereas means and standard deviations were calculated for continuous variables. A confirmatory factor analysis (CFA) was estimated using MPlus to evaluate the factor structure of the CAMI. As the 40-items of the SF-12 were measured on an ordinal scale, a weighted least squares with mean- and variance-adjusted (WLSMV option in MPlus) estimation was used to model the polychoric correlation matrix (Categorical option in MPlus). The following fit indices were utilized to compare the overall model fit and complexities of the models: i) root mean square error of approximation (RMSEA), ii) comparative fit index (CFI), iii) Tucker-Lewis index (TLI), iv) Standardized Root Mean Square Residual (SRMR). Both CFI and TFI values range from 0 to 1, with higher values representing better fit. CFI values above 0.95 and TLI values above 0.90 are considered to be of excellent fit [25]. With regards to RMSEA, values below 0.08 indicate moderate fit, while values of 0.05 or less indicate close fit to the observed data [26]. Standardized root mean squared residual (SRMR) values were also evaluated, which indicates acceptable fit when values are smaller than 0.08, and good fit when values are smaller than 0.05 [25, 26]. Subsequent follow-up analyses were conducted with SPSS based upon the factor structure of final model of the CFA. Linear mixed models (LMM) were employed as it could account for missing data, individual heterogeneity, and longitudinal measures within the same individual. To examine each CAMI dimension, four LMMs were conducted. The ‘time’ variable (0 = pre-intervention, 1 = post-intervention, 2 = 3-months follow-up) was included in each model as both a random and fixed effect to adjust for the overall and individual variations in the dimension scores over time. Linear and quadratic effects, along with interaction terms and covariates, were tested in the model as fixed parameters. Firstly, each model was conducted unconditionally (i.e. without any covariates) to examine the CAMI dimension scores across the time points. Next, sociodemographic variables (i.e. age, gender, ethnicity, nationality, having friends/family with a mental illness, and having experience within the mental health field) were factored in as time-invariant covariates. Subsequently, interactions between linear and quadratic effect and covariates were explored in order to account for any potential effect these interactions might have on the rate of change in CAMI scores over time. Tested interaction effects that were not significant and did not significantly improve model fit based on -2LogLikelihood (−2LL), Akaike Information Criterion (AIC), and Bayesian Information Criterion (BIC) values were not included in the final model.

Effect sizes for each dimension were calculated to compare pre-intervention scores to post-intervention and 3-months follow-up using the formula: $Cohe{n}^{\prime }s\ d=\frac{\left( MeanTime2- MeanTime1\right)}{Pooled\ S.D.}$.

## Results

### Sample characteristics

The characteristics of the sample are presented in Table 1. A total of 390 students participated, the majority were female (60.3%), Chinese (82.8%), and held Singaporean/PR citizenship (82.1%). In addition, 22.1% of the sample reported having “past experience within the mental health field”, while 42.6% of the sample had a close friend(s) or family member(s) with a mental illness.

**Table 1:** Sociodemographic characteristics of the sample

	Pre- and Post-intervention (*n* = 390)		3-month follow-up (*n* = 324)	
	n	%	n	%
Gender				
Female	235	60.3	197	60.8
Male	155	39.7	127	39.2
Ethnicity				
Chinese	323	82.8	272	84.0
Others	67	17.2	52	16.0
Nationality				
Singaporean/PR	320	82.1	270	83.3
Student Visa	70	17.9	54	16.7
Family or friends with mental illness				
Yes	166	42.6	134	41.4
No	224	57.4	190	58.6
Past experience in mental health field				
Yes	86	22.1	75	13.3
No	301	77.2	247	86.7
	Mean	S.D.	Mean	S.D.
Age (in years)	22.28	2.26	22.25	2.24

### Confirmatory factor analysis

Initially, a 4-factor model (Authoritarianism, Benevolence, Social Restrictiveness, and Community mental health ideology) based upon Taylor and Dear’s factor structure was tested [27]. Unfortunately, this model failed to converge due to a non-positive definite covariance matrix. Upon further examination, it was revealed that this issue was caused by high dependency between the specified Authoritarianism and Social Restrictiveness latent factors, which suggested that these two factors were statistically indistinguishable from one another. Furthermore, the original developers state in their article that their factor analysis revealed that “Authoritarianism and social restrictiveness are approximately equally correlated with the first factor and, to a lesser extent, with the fourth factor. This provides some evidence that these two scales perhaps represent a single dimension. They are treated separately, however, in the subsequent analyses.” [27]. However, in this present paper, a 3-factor model in which the items corresponding to the original Authoritarianism and Social Restrictiveness factors were subsumed into a single dimension was tested. This model had decent overall fit (RMSEA = 0.06, CFI = 0.92, TLI = 0.92, SRMR = 0.06), but five items (items 4, 13, 23, 26, 29) had factor loadings from 0.001 to 0.28, and were thus removed in subsequent models. The final model had minor improvement in fit indices (RMSEA = 0.06, CFI = 0.93, TLI = 0.93, SRMR = 0.06). Standardized factor loadings for this final model are displayed in Table 2. Cronbach’s α values were calculated and the respective items of each factor were summed for three scale scores: i) Authoritarianism (17-items, α = 0.84), ii) Benevolence (9-items, α = 0.78), iii) Community Mental Health Ideology (9-items, α = 0.83).

**Table 2:** Standardized factor loadings for the final three-factor model (Model 3) for the CAMI scale

CAMI Items	Estimate
Authoritarianism	
One of the main causes of mental illness is a lack of self-discipline and will power. ^┼^	0.58
The best way to handle the mentally ill is to keep them behind locked doors.	0.71
There is something about the mentally ill that makes it easy to tell them from normal people.	0.48
As soon as a person shows signs of mental disturbance, he should be hospitalized.	0.48
Mental patients need the same kind of control and discipline as a young child.	0.55
Mental illness is an illness like any other. ^┼^	0.54
The mentally ill should not be treated as outcasts of society. ^┼^	0.77
Virtually anyone can become mentally ill. ^┼^	0.52
The mentally ill should not be given any responsibility.	0.53
The mentally ill should be isolated from the rest of the community.	0.63
A woman would be foolish to marry a man who has suffered from mental illness, even though he seems fully recovery	0.66
I would not want to live next door to someone who has been mentally ill.	0.72
Anyone with a history of mental problems should be excluded from taking public office.	0.59
The mentally ill should not be denied their individual rights. ^┼^	0.66
No one has the right to exclude the mentally ill from their neighbourhood. ^┼^	0.56
The mentally ill are far less of a danger than most people suppose. ^┼^	0.46
Most women who were once patients in a mental hospital can be trusted as baby sitters. ^┼^	0.44
Benevolence	
The mentally ill have for too long been the subject of ridicule.	0.48
More tax money should be spent on the care and treatment of the mentally ill.	0.56
We need to adopt a far more tolerant attitude toward the mentally ill in our society.	0.68
We have the responsibility to provide the best possible care for the mentally ill.	0.66
The mentally ill do not deserve our sympathy. ^┼^	0.53
The mentally ill are a burden on society. ^┼^	0.62
Increased spending on mental health services is a waste of tax dollars. ^┼^	0.78
There are sufficient existing services for the mentally ill. ^┼^	0.41
It is best to avoid anyone who has mental problems. ^┼^	0.80
Community Mental Health Ideology	
Residents should accept the location of mental health facilities in their neighbourhood to serve the needs of the local community	0.77
As far as possible mental health services should be provided through community-based facilities.	0.49
Locating mental health services in residential neighbourhoods does not endanger local residents.	0.71
Residents have nothing to fear from people coming into their neighbourhood to obtain mental health services.	0.69
Mental health facilities should be kept out of residential neighbourhoods. ^┼^	0.72
Local residents have good reason to resist the location of mental health services in their neighbourhood. ^┼^	0.61
Having mental patients living within residential neighbourhoods might be good therapy, but the risks to residents are too great^. ┼^	0.71
It is frightening to think of people with mental problems living in residential neighbourhoods. ^┼^	0.77
Locating mental health facilities in a residential area downgrades the neighbourhood. ^┼^	0.66
Latent Factor Correlation	
Authoritarianism with Benevolence	−0.95
Authoritarianism with Community Mental Health Ideology	−0.90
Benevolence with Community Mental Health Ideology	0.89
Removed items	
The best therapy for many mental patients is to be part of a normal community.	–
Less emphasis should be placed on protecting the public from the mentally ill.	–
Mental patients should be encouraged to assume the responsibilities of normal life.	–
Our mental hospitals seem more like prisons than like places where the mentally ill can be cared for.	–
Mental hospitals are an outdated means of treating the mentally ill.	–

^┼^Indicates that the item was reverse scored

### Authoritarianism scores across time

**Fig. 1:**
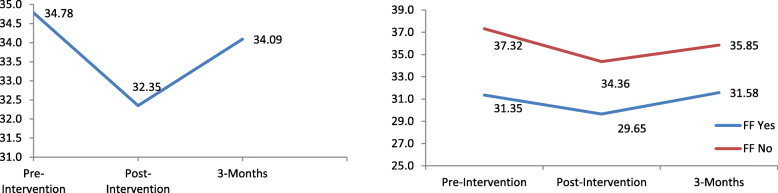
Left - Mean Authoritarianism scores across time. Right - Visualization of the interaction between linear effect and having family or friends with a mental illness on Authoritarianism scores

As indicated in Table 3, results of the unconditional LMM indicated that when compared to pre-intervention, there was a significant decrease in Authoritarianism scores from pre- to post-intervention (*p* <  0.001), and a significant increase in scores from Post-intervention to 3-months follow-up (*p* <  0.001*)*. Effect size of the intervention on authoritarianism scores was small at post-intervention (*d* = 0.32). There were no significant differences between scores at pre-intervention and at 3-months (*p* = 0.07). Adjusting for sociodemographic correlates, the results of the LMM (Table 4) indicated significant linear (B = − 4.85, 95% CI: −5.87, −3.83, *p* <  0.001) and quadratic effects (B = 2.10, 95% CI: 1.63, 2.58, *p* <  0.001), representing a decrease in scores immediately after the intervention, but was followed by an increase in scores at 3-months follow-up (Fig. 1).

**Table 3:** Authoritarianism, Benevolence, Social restrictiveness, and Community mental health ideology scores at pre-intervention, post-intervention, and at 3-months follow up

	Pre-intervention			Post-intervention			3 months			Effect size (Cohen’s *d*^a^)		*p* values^b^		
	n	mean	S.D.	n	mean	S.D.	n	mean	S.D.	Post-intervention	3-month follow-up	Pre vs Post	Pre vs 3 months	Post vs 3 months
Authoritarianism	387	34.78	7.80	388	32.35	7.56	324	34.09	8.23	0.32	0.09	**< 0.001**	0.07	**< 0.001**
Benevolence	380	36.21	4.37	388	36.93	4.16	324	36.22	4.19	0.17	0.002	**< 0.001**	0.91	**< 0.001**
Community Mental Health Ideology	388	35.33	4.84	389	37.41	4.80	324	36.01	4.96	0.43	0.14	**< 0.001**	**0.01**	**< 0.001**

^a^ $$ Cohe{n}^{\prime }s\ d=\frac{\left( MeanTime2- MeanTime\right)}{Pooled\ S.D} $$

^b^ pairwise comparison of respective scores in unconditional linear mixed models, bold values denotes statistically significant values

**Table 4:** Estimates of Linear Mixed Models examining the effect of the intervention on subscales of the Community Attitudes Toward the Mentally Ill scale

	Authoritarianism			Benevolence			Community Mental Health Ideology		
	B	95% CI	*p*	B	95% CI	*p*	B	95% CI	*p*
Time	**−4.85**	-5.87 − -3.83	**< 0.001**	**1.51**	0.97–2.05	**< 0.001**	**3.82**	3.16–4.49	**< 0.001**
Time^2^	**2.10**	1.63–2.58	**< 0.001**	**− 0.63**	− 0.90 – − 0.35	**< 0.001**	**−1.67**	−2.00 – −1.33	**< 0.001**
Age (Years)	0.18	− 0.13 – 0.49	0.25	− 0.03	− 0.21 – 0.14	0.71	0.08	− 0.12 – 0.27	0.43
Gender									
Female	ref			ref			ref		
Male	1.15	−0.32 – 2.61	0.12	−0.79	−1.61 – 0.03	0.60	**− 1.19**	−2.10 – − 0.27	**0.01**
Ethnicity									
Chinese	ref			ref			ref		
Others	0.01	−1.79 – 1.81	0.99	0.88	−0.13 – 1.89	0.09	0.66	−0.47 – 1.79	0.25
Nationality									
Singaporean/PR	ref			ref			ref		
Student Visa	1.73	−0.06 – 3.53	0.58	−0.57	−1.58 – 0.44	0.27	**−1.23**	−2.35 – − 0.11	**0.03**
Family or friends with mental illness									
No	ref			ref			ref		
Yes	**−5.10**	−6.51 – −3.68	**< 0.001**	**2.33**	1.52–3.13	**< 0.001**	**2.46**	1.58–3.35	**< 0.001**
Past experience in mental health field									
No	ref			ref			ref		
Yes	**−3.33**	−4.92 – −1.75	**< 0.001**	**1.38**	0.49–2.27	**0.002**	**1.40**	0.41–2.40	**0.01**
Interaction terms									
Time* Family or friends with mental illness	**0.85**	0.18–1.53	**0.01**						
Time^2^ * Family or friends with mental illness				**−0.34**	−0.52 – −0.16	**< 0.001**	**−0.22**	−0.45 – − 0.002	**0.048**

B – unstandardized regression coefficient; 95% CI: 95% confidence interval of B

Bold print denotes statistically significant B value

Individuals who had friends/family with mental illness (B = − 5.10, 95% CI: − 6.51, − 3.68, *p* <  0.001) and had past experience in a mental health field (B = − 3.33, 95% CI: − 4.92, − 1.75, *p* <  0.001) had lower scores on the Authoritarianism scale than those without. A significant interaction effect was found between the linear effect and having friends/family with mental illness (B = 0.85, 95% CI: 0.18, 1.53, *p* = 0.01). A smaller decrease in authoritarianism scores from pre- to post-intervention was found among those with friends/family with a mental illness, as compared to their counterparts (Fig. 1).

### Benevolence scores across time

**Fig. 2:**
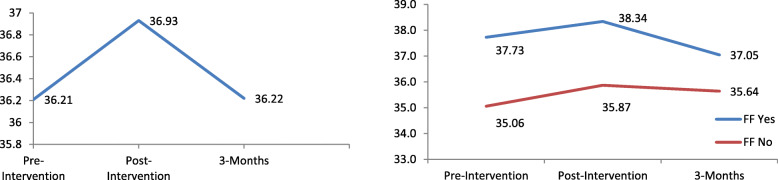
Left - Mean Benevolence scores across time. Right - Visualization of the interaction between quadratic effect and having family or friends with a mental illness on Benevolence scores

The unconditional model indicated a significant increase in benevolence scores at post-intervention (*p* <  0.001) with a small effect size (*d* = 0.17) when compared to pre-intervention scores. There were, however, no significant change in scores at 3-months follow-up. Although, when compared to scores at post-intervention, there was a significant decrease in scores at 3-months follow-up (*p* <  0.001). After controlling for sociodemographic covariates and interaction terms, the results of the LMM (Table 3) revealed a significant linear (B = 1.51, 95% CI: 0.97, 2.05, *p* <  0.001) and quadratic effect (B = − 0.63, 95% CI: − 0.90, − 0.35, *p* <  0.001) indicating that there was an increase in benevolence scores at post-intervention, and a significant decrease in scores from post-intervention to scores at the 3-months follow-up (Fig. 2).

Having family/friends with a mental illness was associated with higher benevolence scores (B = 2.33, 95% CI: 1.52, 3.13, *p* <  0.001). In addition, having experience in a mental health field was associated with significantly higher benevolence scores (B = 1.38, 95% CI: 0.49, 2.27, *p* = 0.002) as well. A significant interaction was found between the quadratic effect and having friends/family with mental illness (B = − 0.34, 95% CI: − 0.52, − 0.16, *p* <  0.001). A greater decrease in benevolence scores from post-intervention to 3-months follow-up was observed among those with friends/family with mental illness compared to their counterparts (Fig. 2).

### Community mental health ideology scores across time

**Fig. 3:**
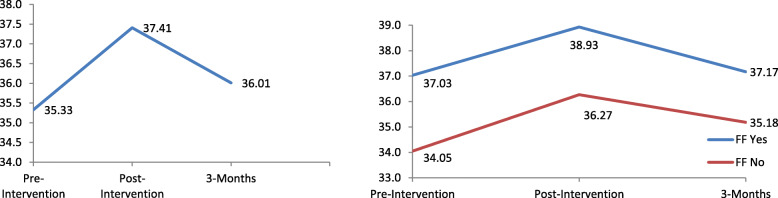
Left - Mean Community mental health ideology scores across time. Right – Visualization of interaction between quadratic effect and having family or friends with a mental illness on Community mental health ideology scores

The unconditional LMM indicate that when compared to the pre-intervention scores, both post-intervention (*p* <  0.001) and at 3-months follow-up (*p* = 0.01) were significantly higher. The effect size of the intervention was small at both time points (*d* = 0.43; 0.14, respectively). Results also indicated a significant increase in scores from post-intervention to 3-months follow-up (*p* <  0.001*)*. After adjusting for sociodemographic covariates, the linear (B = 3.82, 95% CI: 3.16, 4.49, *p* <  0.001) and quadratic (B = − 1.67, 95% CI: − 2.00, − 1.33, *p* <  0.001) effects were significant, indicating an increase at post-intervention and a decrease at 3-months follow-up (Fig. 3).

Of the sociodemographic correlates analysed across time, males had significantly lower community mental health ideology scores than females (B = − 1.19, 95% CI: − 2.10, − 0.27, *p* = 0.01). Individuals who were on student visas also had significantly lower community mental health ideology scores than Singaporeans/PRs (B = − 1.23, 95% CI: − 2.35, − 0.11, *p* = 0.03). In contrast, having friends/family with a mental illness (B = 2.46, 95% CI: 1.58, 3.35, *p* <  0.001) and past experience in a mental health field (B = 1.40, 95% CI: 0.41, 2.40, *p* = 0.01) was associated with higher community mental health ideology scores. A significant interaction effect was found between the quadratic effect and having friends/family with a mental illness (B = − 0.22, 95% CI: − 0.45, − 0.002, *p* = 0.048). A greater decrease in community mental health ideology scores in individuals who have friends/family with mental illness from post-intervention to the 3-months follow-up, as compared to their counterparts, was observed (Fig. 3).

## Discussion

This paper examined the effectiveness of the ARTEMIS intervention on the 3-factor model of the community attitudes to mental illness (CAMI) scale towards depression. Significant shifts in attitudes towards depression was observed across the timepoints (pre-intervention, post-intervention, and 3-months follow-up). When comparing baseline and post-intervention scores, favourable attitudes (i.e. lower scores on authoritarianism, and higher scores on benevolence and community mental health ideology) were attained in all 3 factors of the CAMI. It is established that the lack of mental health literacy (i.e. knowledge) is closely associated with greater stigmatising attitudes towards mental illness [28–30]. This is in line with findings of previous studies that incorporate education as a component of their respective anti-stigma intervention to address the lack of knowledge [17, 20, 31]. For instance, the education component of the ARTEMIS intervention incorporates a biopsychosocial approach of depression for university students in Singapore. In a study by Han et al., they found that biological attribution of depression saw greater improvements in help-seeking intentions, whereas de-stigmatisation information helped reduced negative appraisals of people with depression [32]. Aside from the education component, social contact via the sharing session by a person with lived experience could have contributed to the shifts in attitudes as well [33]. According to the intergroup contact theory, as part of the four optimal conditions, equal status needs to be established to reduce prejudice between groups [34]. Therefore, the social contact component of the intervention could have helped breach the status gap between groups, which could have led to alleviated levels of anxieties surrounding depression as well as an increased sense of empathy towards PMI [33, 34]. Wood and Wahl recruited undergraduate students for a mental health education programme known as In Our Own Voice (IOOV) [31]. When Wood and Wahl compared their participants who attended IOOV against their control group, they found significant improvements in attitudes towards PMI.

On the other hand, significant findings were found between post-intervention and at 3-months follow-up as well; although there is a reversal of the results which had previously shifted. The analysis indicated that authoritarianism scores increased, whereas benevolence and CMHI scores decreased when comparing post-intervention and 3-months follow-up timepoint. However, when comparing mean scores of the 3 factors between baseline and at 3-months follow-up, it seems to suggest that scores at the follow-up reverted to baseline scores. The strength of one’s attitude could be a possible explanation for the reverted scores. Attitude strength is a notion that describes how strongly one feels about a specific person, object or concept [35]. Petty et al. had established a correlation between attitude strength and the likeliness of being persuaded – where the stronger the attitude the individual holds, the more resistant it is to change. It could be reasoned that participants possessed strong attitudes towards people with depression prior to the intervention. Supplementing the fact that the study took a convenience sampling approach to recruitment, a potential for self-selection bias might be in play, thus the participating university students might already have vested interest in the topic. Although being resistant to change due to the strength of one’s attitude does not account for the immediate shift in favourable attitudes from baseline to post-intervention. This trend indicates that the ARTEMIS intervention was able to elicit a short-term favourable shift in attitudes towards depression but was not able to achieve any lasting effects. As reported by Thornicroft et al., although short-term improvements in knowledge and attitudes could be observed immediately after an anti-stigma intervention, the effects would weaken over time [36]. To resist changes in attitudes across time, McGuire proposed the inoculation theory, two types of bolstering efforts which would maintain the intervention effects: 1) providing information for active recollection of the session, and 2) subsequent exposure to less complex follow-up sessions [35]. Therefore, future anti-stigma interventions should be mindful of temporal influences and incorporate McGuire’s proposed efforts to maintain lasting and favourable shifts in attitudes following the intervention.

In comparison with other anti-stigma interventions, the ARTEMIS intervention seems to report similar trends. For instance, in Ahuja et al., the combined utilisation of education and social contact in their anti-stigma intervention for college students (18–21 years) in India observed favourable shifts in CAMI scores immediately after the intervention [20]. Their follow-up period of 1-week also saw sustained favourable attitudes towards PMI, although it would be important to note that compared to the ARTEMIS intervention their follow-up period was much shorter. Friedrich et al. also reported similar results, where medical students (mean age = 23.5) underwent the Education Not Discrimination (END) component of the Time to Change anti-stigma programme to reduce mental illness stigma among medical professionals and trainees [21]. Immediately after the intervention that included both education and social contact components, favourable shifts in stigma-related attitudes were observed. However, unlike Ahuja et al. but more similar to the current study, they saw no sustained effects of the intervention except for a single item on the CAMI “There is something about people with mental illness that makes it easy to tell them from normal people”. This study, thus, evinced that the combined components of education and social contact could result in immediate favourable shifts in attitudes among university students in Singapore.

The study found that gender, nationality, having experience in a mental health field, as well as close social contact (i.e. friends and family members with mental illness) could influence the CAMI factors. Male students were less supportive of the community mental health ideology dimension towards people with depression when compared to female students. In a study in Spain which was conducted among participants aged 14–18 years, similar gender differences were detected; and Vila-Badia et al.’s findings align with the current paper which found that male students had lower community mental health ideology scores than female students [37]. Savrun et al. postulated that female students possessed more “optimistic values” towards treatment for PMI than male students [38]. Individuals who were on student visas were less supportive of community mental health ideology. As attempts at deinstitutionalisation occur gradually, it is possible that traditional psychiatric mental health care (i.e. asylums and institutionalisation) is still pervasive among the country of origin which the students with visas are from; and thus more familiar to them [39, 40]. Regarding having experience in the mental health field, the results indicated a favourable shift across the CAMI factors. This supports findings from other studies that looked at healthcare professionals and volunteers, which found lower stigma scores after exposure and experience in a mental healthcare environment [34, 41, 42].

Across the 3 CAMI factors, having a close social contact with PMI resulted in more favourable mean scores than those without. The current findings are consistent with previous studies that suggest individuals with a close social contact with PMI reported lower levels of stigmatising attitudes [43, 44]. In contrast, the linear effect observed a smaller decrease in authoritarianism scores among university students with close social contact than those without. Perhaps they are more aware of the realities of caring or befriending a PMI, and as such were less influenced by the intervention [35]. Quadratic effects were observed for both benevolence and CMHI, indicating a greater decrease in scores from post-intervention and at 3-months follow-up. External influences such as recent negative interaction with PMI during the 3-months intermission could provide a plausible explanation for this observed shift for benevolence. Nevertheless, it would be important to highlight that participants with close social contact continue to show more favourable attitude mean scores across the 3 CAMI factors than their counterparts (Fig. 1-3).

### Limitations

A limitation of this study is that the participants were university students recruited from a single local university, and thus would not be considered representative of all university students as there could be unique university cultural influences at play that were not accounted for. As the university students were voluntary participants, baseline scores could be skewed towards more favourable attitudes because of motivation and interest towards the topic as mentioned above. Moreover, seeing as the students come from a diverse background of disciplines and courses, the concept of mental health and stigma might have been a topic covered in certain courses which was not accounted for in this study. Furthermore, not reporting the response rate is a limitation of the convenience sampling method adopted in this study.

## Conclusion

To conclude, the anti-stigma intervention towards depression observed promising short-term favourable shifts across the CAMI factors. After adjusting for sociodemographic correlates, short-term effects of the anti-stigma intervention were significant. However, the intervention did not result in any sustained effects, possibly due to cognitive biases, attitude strength, and the lack of additional sessions of the intervention. Sociodemographic correlates such as gender, nationality, and experience in a mental health field as well as having a close friend/family member with mental illness were identified as significant correlates. Future research exploring whether additional and less complicated follow-up sessions would have any sustained long-term effect on stigmatising attitudes should be considered. Perhaps future interventions could target areas of mental health which is most prevalent in the community or relevant to the environment and slowly integrate more topics opposed to a general “mental illness” approach. Additionally, stigma is highly influenced by the culture of the community, conducting qualitative studies into the specific culture of the university and of the students’ respective community could evoke nuanced insights of the stigma faced. Other possible avenues to explore include the comparisons of anti-stigma interventions focusing on 1) different mental illnesses; 2) other local universities; and 3) other populations.

## Data Availability

Data supporting the findings is available upon request. Please contact the Principal Investigator of the study, Professor Chong Siow Ann at siow_ann_chong@imh.com.sg, for data availability.

## Abbreviations

ARTEMIS: Advancing Research To Eliminate Mental Illness Stigma
Q&A: Questions and Answers
CAMI: Community Attitudes towards Mental Illness
CFA: Confirmatory Factory Analysis
CMHI: Community Mental Health Ideology
PMI: Person/People with Mental Illness
MDD: Major Depressive Disorder
GAD: Generalised Anxiety Disorder
WHO: World Health Organization
WLSMV: Weighted Least Square Mean and Variance Adjusted
RMSEA: Root Mean Square Error of Approximation
TLI: Tucker-Lewis Index
SRMR: Standardised Root Mean Residual
LMM: Linear Mixed Models
-2LL: 2LogLikelihood
AIC: Akaike Information Criterion
BIC: Bayesian Information Criterion
